# Melatonin attenuated the brain damage and cognitive impairment partially through MT2 melatonin receptor in mice with chronic cerebral hypoperfusion

**DOI:** 10.18632/oncotarget.20382

**Published:** 2017-08-22

**Authors:** Tzu-Hsien Tsai, Cheng-Jei Lin, Sarah Chua, Sheng-Ying Chung, Cheng-Hsu Yang, Meng-Shen Tong, Chi-Ling Hang

**Affiliations:** ^1^ Division of Cardiology, Department of Internal Medicine, Kaohsiung Chang Gung Memorial Hospital and Chang Gung University College of Medicine, Kaohsiung, Taiwan; ^2^ Center for Translational Research in Biomedical Sciences, Kaohsiung Chang Gung Memorial Hospital and Chang Gung University College of Medicine, Kaohsiung, Taiwan

**Keywords:** brain damage, chronic cerebral hypoperfusion, melatonin

## Abstract

**Background:**

Vascular cognitive impairment (VCI) is a spectrum of cognitive impairment caused by various chronic diseases including aging, hypertension, and diabetes mellitus. Oxidative and inflammatory reactions induced by chronic cerebral hypoperfusion (CHP) are believed to cause VCI. Melatonin is reported to possess anti-oxidation and anti-inflammation effects. This study was designed to investigate the effect and mechanisms of melatonin in CHP mice model.

**Results:**

The behavioral function results revealed that CHP mice were significantly impaired when compared with the control. Melatonin improved the cognitive function, but the addition of MT2 receptor antagonist reversed the improvement. The IHC staining showed melatonin significantly improved WM lesions and gliosis in CHP mice. Again, the addition of MT2 receptor antagonist to melatonin worsened the WM lesion and gliosis. Similar results were also found for mRNA and protein expressions of oxidative reaction and inflammatory cytokines.

**Materials and Method:**

Forty C57BL/6 mice were divided into four groups: Group 1: sham control; Group 2: CHP mice; Group 3: CHP with melatonin treatment; Group 4: CHP-melatonin and MT2 receptor antagonist (all groups *n* = 10). Working memory was assessed with Y–arm test at day-28 post-BCAS (bilateral carotid artery stenosis). All mice were sacrificed at day-30 post-BCAS. The immunohistochemical (IHC) staining was used for white matter (WM) damage and gliosis. The expression of mRNA and proteins about inflammatory and oxidative reaction were measured and compared between groups.

**Conclusions:**

Partially through MT2 receptor, melatonin is effective for CHP-induced brain damage.

## INTRODUCTION

Vascular cognitive impairment (VCI) is the secondary cause of dementia in the world [1–3]. VCI increases the morbidity, disability, and health care costs in the growing elderly population, and decreases the quality of life and survival rate of patients despite aggressive therapy [1–8]. Aging, chronic hypertension, and diabetes mellitus have been reported associations with the onset of VCI, which is characterized by white matter (WM) lesions [9–11]. These vascular risk factors result in vascular remodeling, which then lead to chronic cerebral hypoperfusion (CHP). CHP, a consequence of chronic hypertension, diabetes mellitus and aging, is a critical factor that leads to the onset of VCI [9–11]. Characterized by blood-brain barrier (BBB) disruption, WM lesions, damage of oligodendrocytes, activated gliosis and cognitive dysfunction, CHP triggers off oxidative reactions and inflammatory processes, resulting in white matter (WM) damage [12–14].

Melatonin is synthesized primarily in the pineal gland, and is involved with the circadian rhythm [15–17]. Several of its actions are mediated via two G-protein-coupled membrane receptors called MT1 and MT2. [15–17] Additionally, melatonin has been shown to be a powerful antioxidant with multifaceted protective capacities against oxidative stress and the organ dysfunction from the enhancement of reactive oxygen species (ROS) generation [15–19]. Previous studies have shown that melatonin therapy remarkably reduces the generation of ROS as well as oxidative stress in animal models of ischemia-reperfusion injury [18, 19]. Based on the anti-oxidative and inflammatory effects of melatonin, the use of melatonin can be projected to offer benefits for CHP-induced vascular-impaired dementia (VID). However, it is unclear whether these effects are caused solely by melatonin, or also partially by the activation of the melatonin MT2 receptor [20–23]. This study was designed to test whether or not melatonin attenuates CHP induced brain damage and MT2 receptors take part in the aforementioned process.

## RESULTS

### The effect of melatonin on cerebral blood flow and working memory in CHP mice

**Figure 1:**
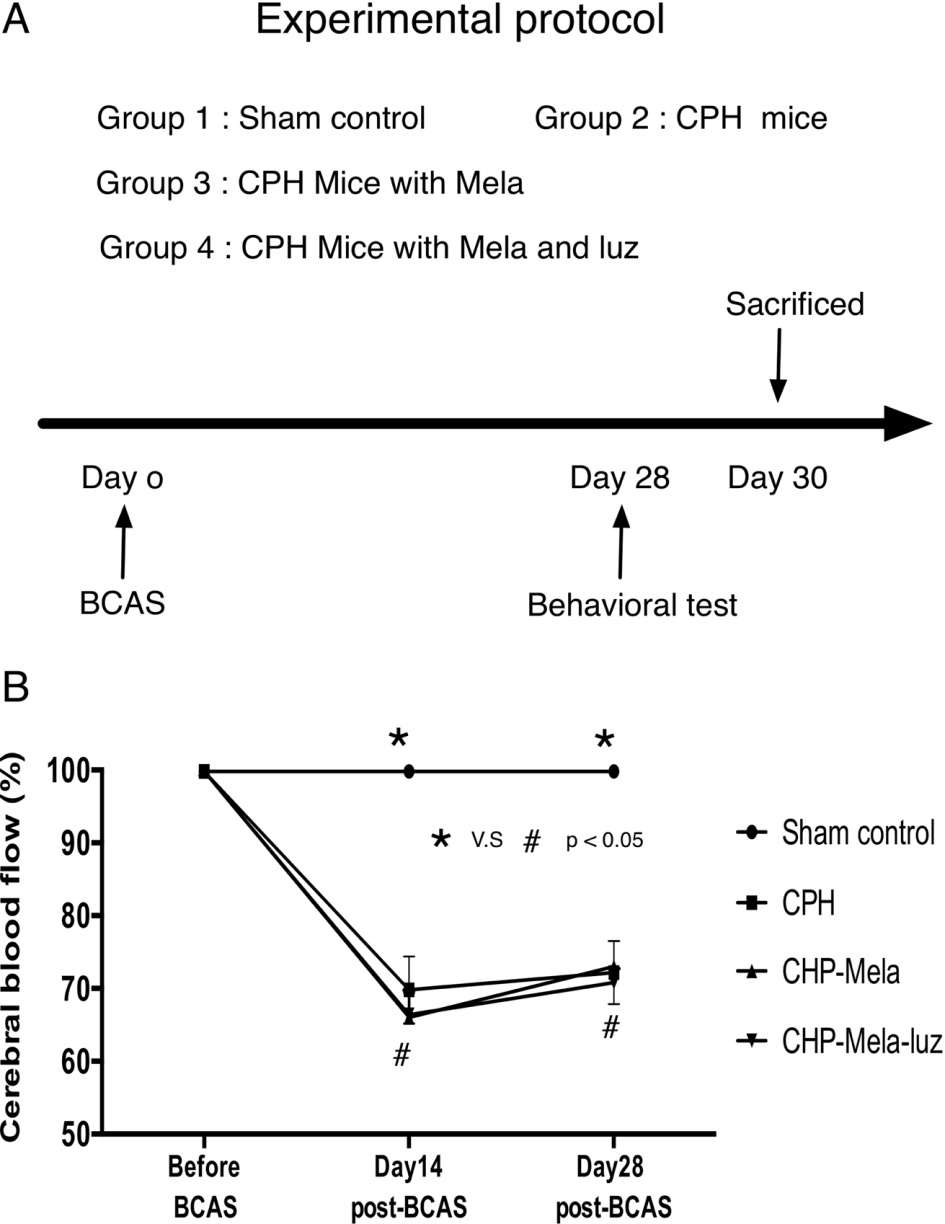
(**A**) The animal experimental protocol. Time course of individual interventional procedure during the study period. The melatonin and luzindole were treated at 24 hours post-bilateral carotid artery stenosis(BCAS) until 30 days post-BCAS. SC = sham control; CHP = cerebral hypoperfusion mice; CHP-Mela = cerebral hypoperfusion mice with melatonin (10 mg/kg/day) treated; CHP-Mela-Luz = cerebral hypoperfusion mice with melatonin(10 mg/kg/day) and luzindole (30 mg/kg/day, ip). (**B**) Laser Doppler flow recordings in different groups. The Laser Doppler analysis of cerebral blood flow(CBF) was performed. Results represent the mean values ± SEM in different groups mice. Group 1 = sham control, Group 2 chronic cerebral hypoperfusion(CHP) mice, Group 3: melatonin treatment in CHP mice, Group 4: addition MT2 antagonist with melatonin in CHP mice. The decrease in CBF at day 30 post-BCAS of approximately 70% was similar between CHP, CHP-Mela and CHP-Mela-Luz mice groups. Different symbols (#, *) indicate significance, *p* < 0.05. All statistical analyses using by repeat two-away ANOVA test, followed by Bonferroni multiple comparison post hoc test.

The study protocol is presented in Figure 1A. The cerebral blood flow (CBF) values from the deceased mice were excluded because extremely low CBF was found in these mice, which was resulted from systemic hypotension. In group 1, the mean CBF after the sham operation varied from 97.0% to 102% of the baseline level throughout the entire experiment. In groups 2, 3, and 4, the CBF values showed a significant decrease to around 65% of the baseline level at day-14 post-BCAS. The CBF values remained relatively low, at 75% of the baseline level, at day-28 post-BCAS (Figure 1B).

**Figure 2:**
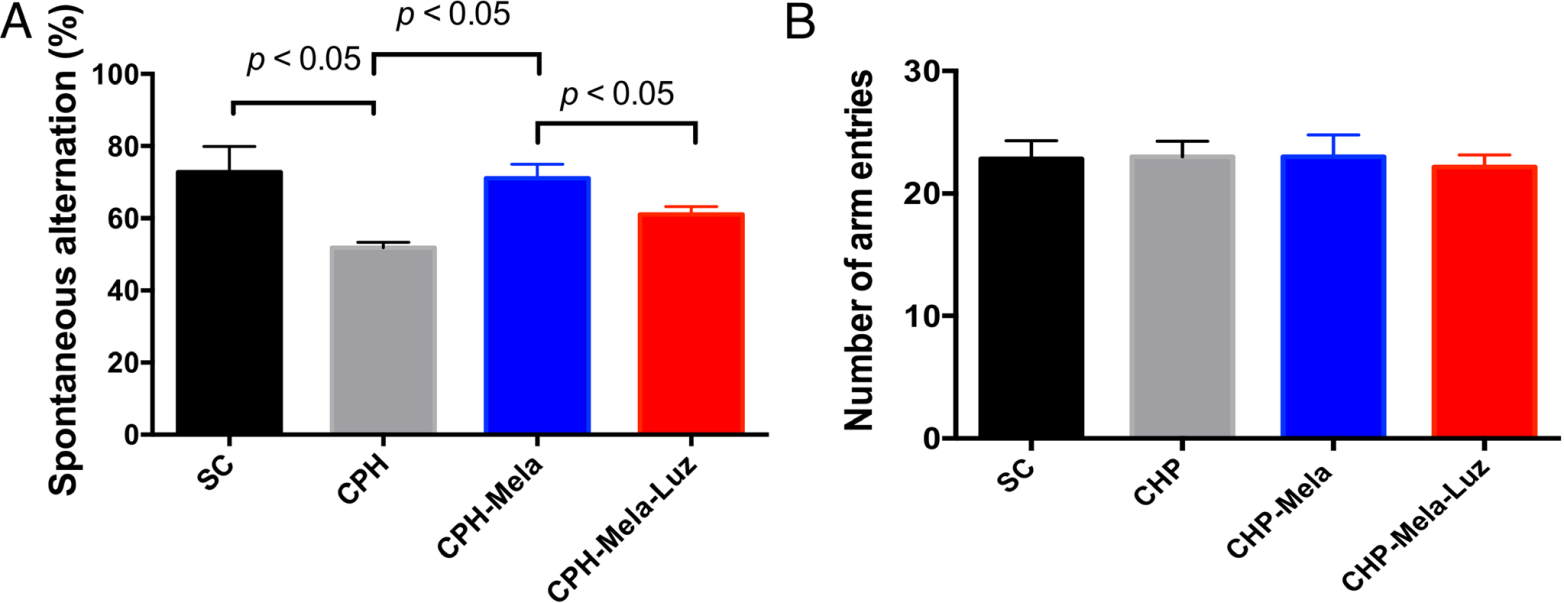
Effects of melatonin and melatonin with MT2 receptor antaginist on spatial working memory in mice post-BCAS at day-28 Spontaneous alternation. Group 1 = sham control, Group 2 chronic cerebral hypoperfusion(CHP) mice, Group 3: melatonin treatment in CHP mice, Group 4: addition MT2 receptor antagonist with melatonin in CHP mice. Values are mean SEM (*n* =10 in each group). Spontaneous alternation ratio was decreased in group 2 than group 1. The treated melatonin increased the Spontaneous alternation ratios in group 3 than group 2. However, the addition MT2 receptor antagonist with melatonin abolished the protective effect of melatonin in group 4. The *p* value < 0.05 indicates significance between two groups. All statistical analyses using one-way ANOVA, followed by Bonferroni multiple comparison post hoc test. Number of arm entries. Group 1 = sham control, Group 2 chronic cerebral hypoperfusion(CHP) mice, Group 3 : melatonin treatment in CHP mice, Group 4 addition MT2 receptor antagonist with melatonin in CHP mice. Values are mean SEM (*n* =10 in each group). There was no significant difference between four groups. All statistical analyses using one-way ANOVA.

As shown in Figure 2, the Y maze (spontaneous alternation) was performed to discriminate the working memories between the 4 groups. In regards to CHP mice, group 2 showed a significant impairment in comparison with group 1 *(P* < 0.05). Treatment with melatonin (group 3) significantly improved the spatial working memory when compared with those without additional treatment. However, the positive effect of melatonin was lessened when the MT2 receptor antagonist was added to melatonin (group 4). There was no significant difference between four groups in numbers of arm entries.

### Effects of melatonin on WM Lesions, and glial activation in CHP Mice

**Figure 3:**
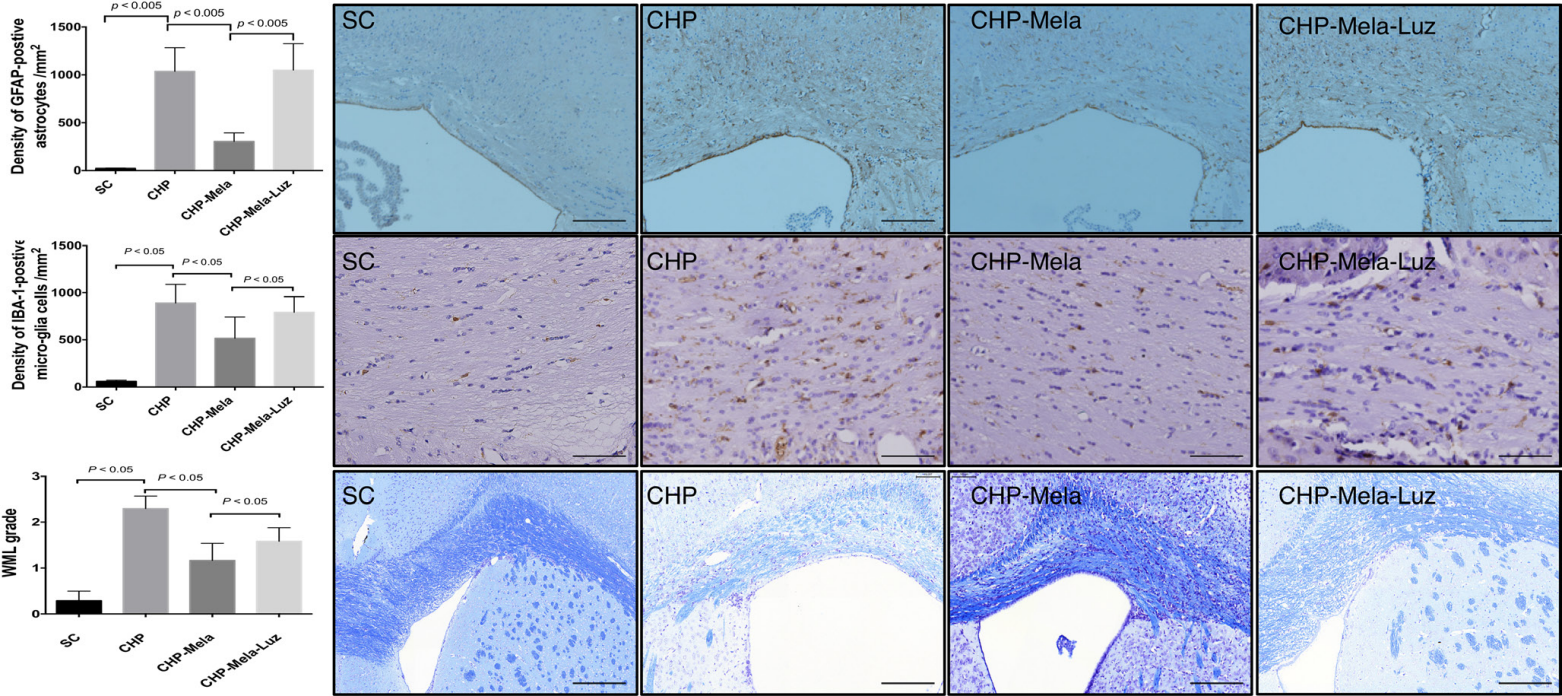
Effects of Melatonin on the activation of astrocytes (**A**) microglia (**B**) and white matter lesion (**C**) in mice with chronic cerebral hypoperfusion. Upper panels: are representative photomicrographs of immunostaining of glial fibrillary acidic protein(GAFA) in the paramedian parts of the corpus callosum. Density of GFAP-positive astrocytes; Scale bar = 200 um. Middle panels: are representative photomicrographs of immunostaining of ionized calcium binding adaptor molecule-1 (IBA-1)-positive microglial cells in the paramedian parts of the corpus callosum. Density of Iba-1-positive microglial cells; Scale bar = 100 um. Lower panel: Immunohistochemical (IHC) images (100×) illustrating white matter lesion (WML) of different severity reflected in the grading; Scale bars in right lower corner represent 200 µm. All statistical analyses using one-way ANOVA, followed by Bonferroni multiple comparison post hoc test. SC = sham control; CHP = cerebral hypoperfusion; CHP-Mela = cerebral hypoperfusion with melatonin (10mg/kg/day,ip); CHP-Mela-Luz = cerebral hypoperfusion with melatonin (10 mg/kg/day,ip) and luzindole (30 mg/kg,ip).

As shown in Figure 3A–3B, the activated microglial cells and astrocytes were significantly increased in CHP mice received BCAS (group 2) as compared with SC (group 1). Besides, the activated microglial cells and astrocytes in CHP mice were significantly attenuated when melatonin was provided (group 3) when compared with mice in group 2. Furthermore, the addition of MT2 antagonist to melatonin treatment raised the number of activated microglial cells in CHP mice (group 4).

The severity grade of WM lesion of the corpus callosum was significantly higher in group 2 when compared with that in group 1. The WM lesion was found significantly reduced when melatonin was offered (group 3). However, the addition of MT2 receptor antagonist to melatonin worsened the WM lesion in group 4 mice (Figure 3C).

### Effects of melatonin on the mRNA expression of inflammatory cytokine and oxidative stress in CHP Mice

**Figure 4:**
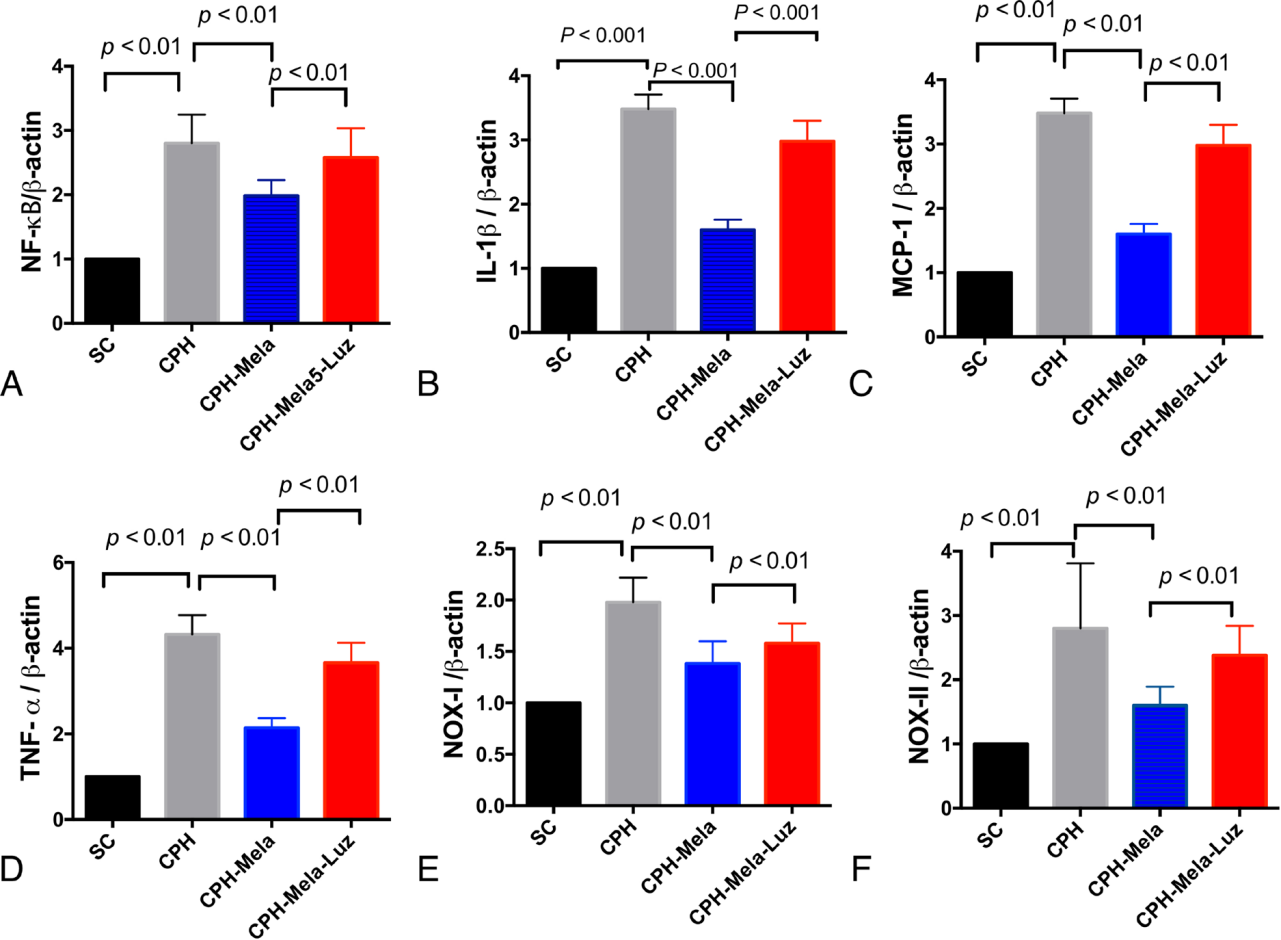
The mRNA expressions of inflammatory and oxidative markers in cerebral cortex by day 30 after chronic cerebral hypoperfusion (CHP) procedure (*n* = 6) (**A**) mRNA expression of IL-1ß; (**D, B**) mRNA expression of tumor necrotic factor (TNF)-α, (**C**) The mRNA expression of monocyte chemoattractant protein (MCP)-1; D) The mRNA expression of NF-kB; (**E**) The mRNA expression of NOX-I; (**F**) The mRNA expression of NOX-II; The *p* < 0.05 indicates statistical significance between two groups. All statistical analyses using one-way ANOVA, followed by Bonferroni multiple comparison post hoc test. SC = sham control; CHP = cerebral hypoperfusion; CHP-Mela = cerebral hypoperfusion with melatonin (10 mg/kg/day,ip); CHP-Mela-Luz= cerebral hypoperfusion with melatonin (10 mg/kg/day,ip) and luzindole (30 mg/kg,ip).

As shown in (Figure 4A–4D), the levels of inflammatory cytokines such as cerebral NF-ƘB, IL-1β, MCP-1, and TNF-α were significantly increased in CHP mice received BCAS (group 2) as compared with SC (group 1). Melatonin treatment significantly attenuated the mRNA expression of these inflammatory cytokines in CHP mice (group 3). However, additions with MT2 antagonist and melatonin comprised the inhibitory effect of melatonin on these mRNA expressions of these inflammatory cytokines in CHP mice (group 4). As shown in Figure 4D–4F), the mRNA expressions of oxidation markers such as NOX-I and NOX-II were significantly increased in CHP mice received BCAS (group 2). The provision of melatonin significantly attenuated the mRNA expressions of these oxidative stress markers in CHP mice, but the reduction of mRNA expressions for these oxidative stress markers were significantly compromised by the addition of MT2 antagonist.

### Effects of melatonin on protein expressions of oxidative stress and DNA damage in CHP mice

**Figure 5:**
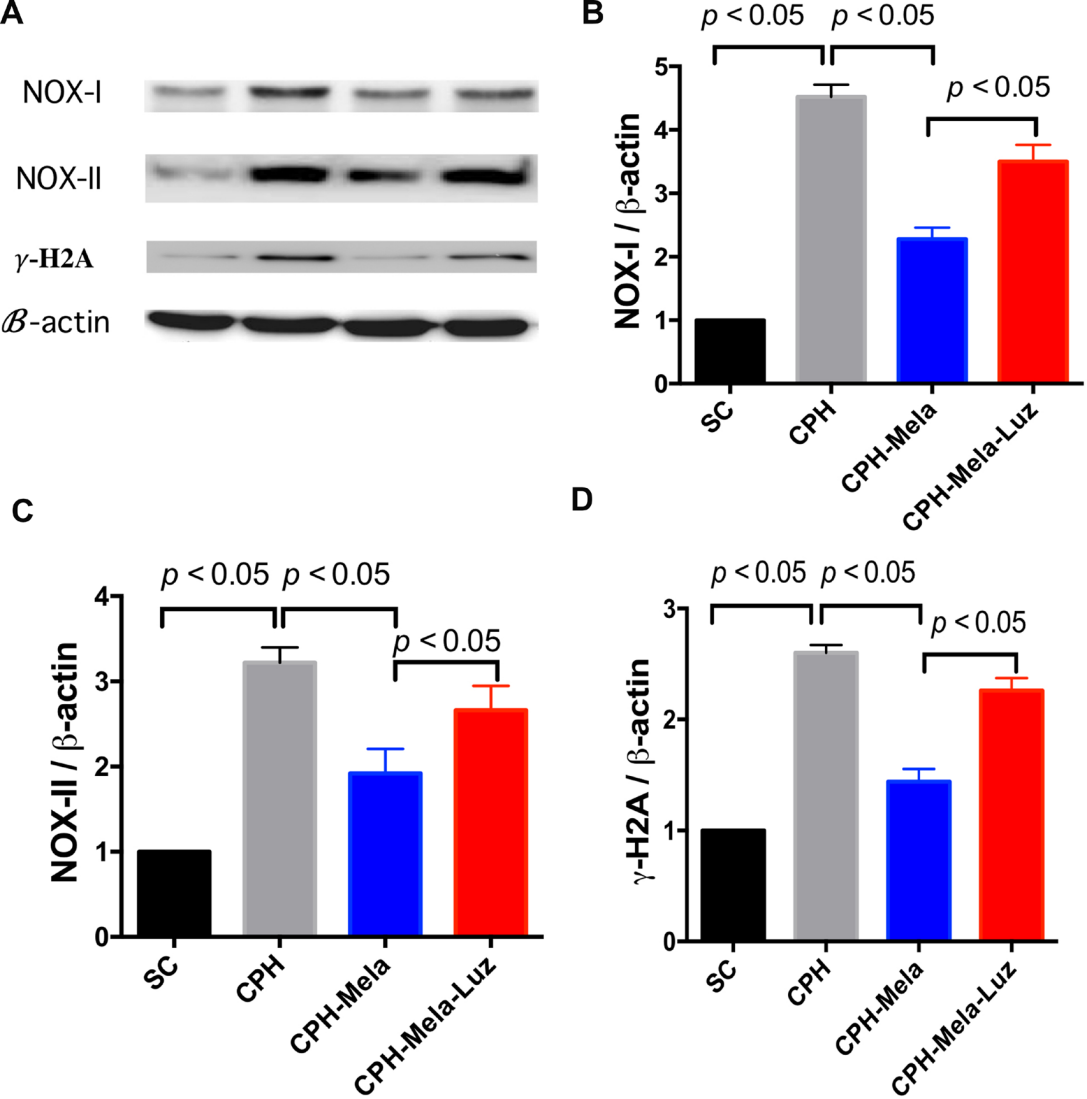
Changes in protein expressions of oxidative and DNA damage markers at 30 days in mice with chronic cerebral hypoperfusion (**A**) Illustration of Western blot results of ROS (i.e., NOX-I and NOX-II) and *r*-H_2_A. (**B**) Protein expression of NOX-I; (**C**) Protein expression of NOX-II;. (**D**) Protein expression of *r*-H_2_A; All statistical analyses using one-way ANOVA, followed by Bonferroni multiple comparison post hoc test. The *p* < 0.05 indicates statistical significance between two groups. SC = sham control; CHP = cerebral hypoperfusion; CHP-Mela = cerebral hypoperfusion with melatonin (10 mg/kg/day,ip); CHP-Mela-Luz = cerebral hypoperfusion with melatonin (10 mg/kg/day, ip) and luzindole (30 mg/kg, ip).

As shown in (Figure 5A–5D), the protein expressions of oxidation markers such as NOX-I and NOX-II induced by CHP were significantly reduced when melatonin was provided. However, this anti-oxidation protective effect of melatonin was reversed when MT2 receptor antagonist was added. The γ-H_2_A is a marker of DNA damage, which helps to represent the condition of brain damage. The protein expressions of γ-H_2_A were significantly increased in CHP mice. The addition of MT2 receptor antagonist to melatonin raised the expression of γ-H_2_A again.

### Effects of melatonin on proteins expression of inflammatory markers in CHP mice

**Figure 6:**
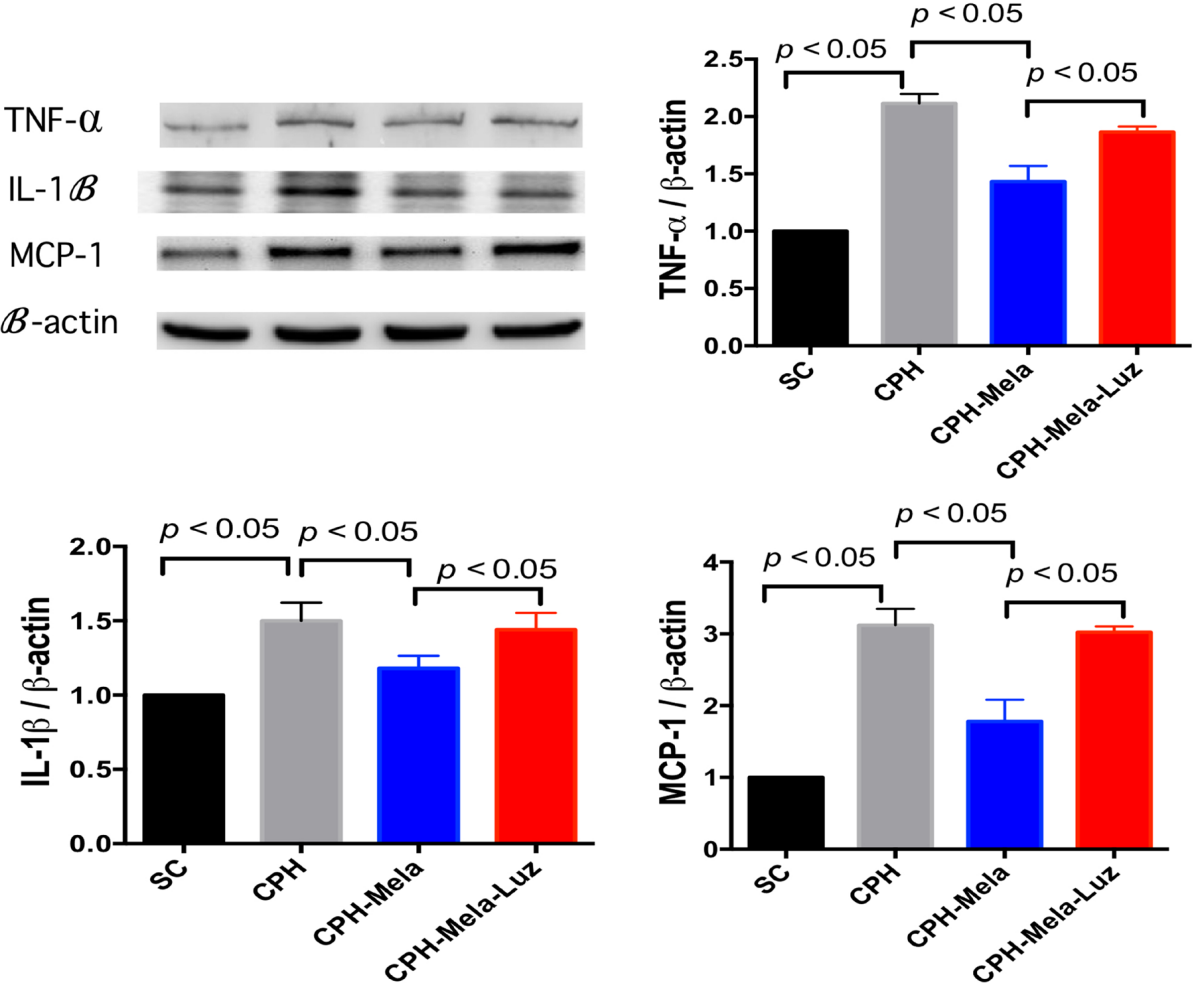
Changes in protein expressions of inflammatory markers at 30 days in mice with chronic cerebral hypoperfusion (**A**) Illustration of Western blot results of inflammatory markers: IL-1ß, T TNF-α and MCP-1. (**B**) Protein expression of IL-1ß; (**C**) Protein expression of TNF-α; (**D**) Protein expression of MCP-1; All statistical analyses using one-way ANOVA, followed by Bonferroni multiple comparison post hoc test. The *p* < 0.05 indicates statistical significance between two groups. SC = sham control; CHP = cerebral hypoperfusion; CHP-Mela = cerebral hypoperfusion with melatonin (10mg/kg/day, ip); CHP-Mela-Luz = cerebral hypoperfusion with melatonin (10 mg/kg/day, ip) and luzindole (30 mg/kg, ip)

As shown in (Figure 6A–6D), the protein expressions of inflammatory markers such as IL-1β, TNF-α and MCP-1 were significantly increased in CHP mice. The protein expressions of IL-1 β, TNF-α and MCP-1 were significantly higher in CHP mice than in SC. Melatonin treatment significantly attenuated these inflammatory protein expressions in CHP mice. However, the addition of MT2 receptor antagonist to melatonin increased these IL-1 β and TNF-α protein expressions again.

## DISCUSSION

This was an investigational study aimed to reveal the therapeutic effects and mechanisms of action for melatonin for the cognitive impairment in CHP mice. The study results demonstrated interesting findings. First, melatonin attenuates both the activation of microglial cells and astrocytes and WM damage were attenuated by melatonin in CHP mice. Second, the cognitive function was improved by melatonin in CHP mice. Third, the action mechanisms of melatonin to improve cognitive function, WM lesions and gliosis were partially mediated through the MT2 receptor pathway which showed anti-oxidative and anti-inflammatory effects. Brain damage and cognitive dysfunction in CHP mice were improved after melatonin treatment. Melatonin might thus be considered as a putative treatment to protect the cognitive function in CHP mice.

### Oxidative stress and inflammation process are major factors in pathogenesis of CHP-induced brain damage

Oxidative stress and inflammation process are reported to be major factors for CHP-induced brain damage and the progression of cognitive dysfunction in the setting of chronic CHP. Bio-markers of oxidative stress such us 8-isoprostaglandin F(2a) (8-isoPGF(2a)) and nitrotyrosine (NT) are obviously elevated in patients with VCI and dementia [24–30]. Inflammatory cytokines such us interleukin (IL)-6, and hs-CRP (high sensitivity- chronic reactive protein) are also elevated in patients with dementia [31–35]. Interestingly, (IL)-6 and hs-CRP are predictors used for cognitive decline. In additional, the decrease of glutathione (GSH) and the elevation of oxidative stress and inflammatory cytokines have been linked to the WM damage and gliosis in animal CHP model [36–41]. The expressions of oxidative stress and inflammation reactions were obviously elevated and correlated with the severity of WM lesion and gliosis in CHP mice in the study. Besides, the brain damage was attenuated and cognitive function improved after oxidative stress and inflammation were inhibited.

### Benefits of melatonin therapy in attenuating CHP brain damage

The study aimed to explore the therapeutic effect and action mechanisms of melatonin to improve cognitive impairment in CHP mice models. The WM lesion and glial cells activation were exhibited in the CHP mice model just as they demonstrated in previous studies [13, 14]. Melatonin was showed to both attenuate the WM lesions and glial cells activation and improve cognitive function in our study. Melatonin is found to possess strong anti-oxidative effect by inhibiting the expression of NADPH oxidases (NOX) in many studies [15, 16, 18, 19, 42]. Among the NOXs, NOX-I and NOX-II play major regulating roles in oxidative stress reactions [15, 16, 18, 19, 42]. Brain damage in CHP mice was effectively attenuated by reducing the oxidative stress in previously published studies [43, 44]. Our study demonstrated that melatonin attenuated the expression of oxidative stress in CHP mice, indicating that melatonin possessed with anti-oxidative effect.

Cerebral hypoxia induced by CHP leads to the increase of inflammation reaction, which plays an important role in the pathogenesis of WM lesion, gliosis and cognitive dysfunction [13, 14]. The expression of inflammatory cytokines (i.e. MCP-1, TNF-α, and IL-1β) was found increased in the CHP mice in our study and this was consistent with the findings in previous reports [13, 14] Melatonin was then found to attenuate the expression of inflammatory cytokines in mRNA and protein levels and improved the WM lesion, gliosis and cognitive function in our study. These results were also consistent with the results in previous reports [12–14]. We therefore conclude that melatonin is effective to attenuate the brain damage induced by CHP through inhibiting oxidative stress and inflammation.

### Protection against brain damage of melatonin may through MT2 receptor pathway

Brain damage induced by CHP would also lead to ischemia which could later result in DNA damage and lipid per-oxidation [43]. The G-protein–coupled melatonin MT1 and MT2 receptors are all expressed in the brain [45]. In addition, melatonin activates the Nrf2-antioxidant responsive element (Nrf2-ARE) pathway in experimental diabetic neuropathy, a subarachnoid hemorrhage model, and ischemic stroke through melatonin receptor [46–48]. It is unclear whether melatonin attenuates the oxidative stress through spontaneous antioxidant effect or the activation of MT2 receptor in CHP model. Melatonin demonstrates cellular protective effect through both anti-oxidative stress and MT2 receptor [49–52]. The reduction of oxidative stress is revealed to alleviate the ischemia-related damage such as stroke and CHP in previous studies [12, 23, 53]. This study was designed to reveal the therapeutic mechanisms of melatonin in CHP mice. Interestingly, we found that the addition of MT2 receptor antagonist to melatonin for CHP mice worsened the WM lesion, glial cell activation and cognitive function. Besides, the comeback of oxidative stress and inflammation in CHP mice was obviously observed after MT2 receptor antagonist was added to melatonin treatment. The protective effect of melatonin in the CHP mice was also reversed by the addition of MT2 receptor antagonist. These findings indicated that MT2 receptor is involved in the anti-oxidative and anti-inflammation effects of melatonin in this CHP mice model. Nevertheless, luzindole (MT2 receptor antagonist) is found to antagonize the protective effect of melatonin [20]. Luzindole is found to possess anti-depression effect as well [22]. Melatonin has also been reported to attenuate ischemic neuronal damage with up-regulation of the MT2 melatonin receptor [21]. Luzindole (MT2 receptor antagonist) significantly reversed the anti-oxidative and anti-inflammation effects of melatonin in CHP mice. These findings suggested that melatonin attenuate the brain damage induced by CHP partially through MT2 receptor to inhibiting oxidative stress and inflammation.

This study has several limitations. First, this study did not investigate the optimal dosage of melatonin for maximizing the brain-protecting effects in this CHP mice model. However, previous studies have demonstrated that the dose of melatonin (10 mg/kg/day) offer adequate protective effects in mice with ischemic organ dysfunction [23, 54]. We therefore followed the dosage they used in previous studies [23, 54]. It proved in the end to be suitable for our study. Second, we didn’t provide the CCH mice with Luzindole only treatment to investigate whether MT2 antagonist were associated with the effects of endogenous melatonin mediated brain damage. However, recent paper found chronic use of MT2 antagonist were involved in the effects of endogenous melatonin to mediate the hippocampal neurogenic process in C57BL/6 mice [55]. This finding provided that MT2 antagonist has an effect on its own, which was contrasts the improvement due to endogenous melatonin. Third, the age of mice is twelve-week-old with a duration of 30 days for cerebral hypoperfusion in the study. Clinical VCI is a long-term progressive disease found in elderly patients. This study did not evaluate the long-term impact of melatonin treatment; it would not be suitable to directly apply these results to a clinical setting. Forth, although the exploration of the therapeutic effects of melatonin was attempted, the precise signaling pathways through melatonin in protecting brain damage in CHP mice model were not completely elucidated.

In conclusion, melatonin offers a substantial therapeutic advantage of protection against CHP-induced brain damage and cognitive dysfunction in a murine model. The results of this study encourage a prospective clinical trial in order to evaluate the therapeutic potential of melatonin in the clinical setting of VCI.

## MATERIALS AND METHODS

### Experimental protocol and bilateral carotid artery surgery

The study protocol is presented in Figure 1A. Twelve-week-old male C57Bl/6J mice (*n =* 40) weighing 25–30 g (Charles River Technology, BioLASCO Taiwan Co. Ltd., Taiwan) were randomized and equally divided into group 1 (sham controls, *n =* 10), group 2 [chronic cerebral hypoperfusion (CHP) with bilateral carotid artery stenosis (BCAS) only, *n =* 10], group 3 [CHP with intraperitoneal (i.p) 10 mg/kg melatonin treatment, beginning 24 hours post BCAS until 30 days post BCAS, *n =* 10], group 4 [CHP with intraperitoneal 10 mg/kg melatonin and 5 mg/kg MT2 receptor antagonist (luzozide), beginning 24 hours post BCAS until 30 days post BCAS, *n =* 10] to determine the therapeutic effect of melatonin on mice with BCAS. Immediately after their working memories were assessed by the Y maze at day-28 post BCAS, mice were sacrificed for a histological and real-time quantitative reverse transcriptase–polymerase chain reaction (RTqPCR) examination at day-30 post-BCAS. All animals were anesthetized by inhalational 3.0% isoflurane and placed supine on a warming pad at 37°C during the BCAS procedure. The chronic cerebral hypoperfusion mice model was induced by BCAS which introducing wire micro coils (Sawane Spring Co, Shizuoka, Japan) with an internal diameter of 0.16 mm at left common carotid artery and 0.18mm around right common carotid artery to result in luminal narrowing (*n =* 30) [12, 13]. Sham-control mice received identical surgical interventions without the coil placement (*n =* 10). All animal experimental procedures were approved by the Institute of Animal Care and Use Committee at Kaohsiung Chang Gung Memorial Hospital and performed in accordance with the Guide for the Care and Use of Laboratory Animals (NIH publication No. 85–23, National Academy Press, Washington, DC, USA, revised 1996).

### Cerebral blood flow measurement

Under deep anesthesia by inhalational 3.0% isoflurane in a 50:50 mixture of oxygen to room air, mice were placed in a prone position on a warming pad at 37°C. The skin overlying the right skull was reflected. Cerebral blood flow was measured using laser Doppler imaging (MoorLD1- 2l; Moor Instruments, Devon, UK). After equilibration to the experimental setup, laser point was fixed perpendicular to the skull at 1 mm posterior and 2.5 mm lateral to the bregma and local cerebral blood flow was obtained. The baseline CBF recordings were obtained before and on day-14 and day-28 after the BCAS. The CBF values were expressed as a percentage of the baseline value.

### The behavioral test: Y arm maze test

Y-maze is designed with 3 arms with 40 cm long, 13.5 cm high and 4 cm wide and locates in a dim room. Each mouse will be placed at the end of an arm and will move through the maze for 10 minutes per session. Spontaneous alternation can be defined as the consecutive entry of a mouse into all three different arms to form a triplet of non-repeated components. The percentage of spontaneous alternation can be calculated as the ratio of actual to possible alterations automatically by the Y-maze system [defined as the number of spontaneous alternation behavior / (the total number of arm entries - 2) × 100] [14].

### Histopathology scoring staining

After BCAS, the mice were anesthetized by inhalational 3.0% isoflurane and perfused transcardially with 0.01 mol/L PBS (pH 7.4), then with a fixative containing 4% paraformaldehyde and 0.2% picric acid in 0.1mol/L phosphate buffer (pH7.4), and stored in 20% sucrose in 0.1 mol/L PBS (pH 7.4). The brains were embedded in paraffin and sliced into 5 μm-thick coronal sections. Kluver-Barrera (KB) staining was used to determine the demyelination changes in different groups. The severity of the WM lesions was graded as normal (grade 0), disarrangement of the nerve fibers (grade 1), the formation of marked vacuoles (grade 2), and the disappearance of myelinated fibers (grade 3) as described elsewhere. [12] For immunohistochemistry, the rest of the coronal blocks were cut into serial sections (10μm thick) in a cryostat and incubated overnight with a rabbit anti–glial fibrillary acidic protein (GFAP) antibody (diluted 1: 1000; DAKO, Denmark) and goat anti-ionized calcium binding adaptor molecule-1 (Iba-1) antibody (1:200, Abcam). Subsequently, these sections were treated with the appropriate biotinylated secondary antibodies (Santa Cruz Biotechnology) and were visualized with 0.01% diaminobenzidine tetrahydrochloride and 0.005% H_2_O_2_ in 50 mmol/L Tris HCl (pH 7.6). The WM lesions were evaluated in corpus callosum. We counted the numerical density of the glial cell nuclei with immunopositive perikarya in a 0.125 mm^2^ area of corpus callosum in 4 animals from each group. We counted the numerical density of the glial cell nuclei with immunopositive perikarya in the white matter, as described [12].

### Real-time quantitative PCR analysis

The mRNA expressions of MCP-1, IL-1β, TNF-α, NF-ϰB, NOX-I and NOX-II in each of the three groups of animals were analyzed with RT-qPCR and compared. RNA was extracted from brain using EuroGold Trifast (EuroClone). Quantitec Reverse Transcription Kit (Qiagen), according to manufacturer’s protocol. Quantitative analysis was performed using SYBR Green 2X PCR Master Mix (Applied Biosystem). Each sample was run in triplicate and normalized to the expression of housekeeping B-actin gene as previously described [12].

### Western blot analysis of brain specimens

Equal amounts (30 μg) of protein extracts from brain of the animals (*n =* 6, for each group) were loaded and separated by SDS-PAGE using 7% or 12% acrylamide gradients. The membranes were incubated with monoclonal antibodies against NAPH oxidase (NOX)-I (1:1500, Sigma), NOX-II (1:500, Sigma), IL-1β (1:1000, proteintech), *r*-H2A (1:1000 Abcam), MCP-1 (1:1000, Millipore), TNF-⍺ (1: 1000, Cell Signaling) were used. Signals were detected with horseradish peroxidase (HRP)-conjugated goat anti-mouse, goat anti-rat or goat anti-rabbit IgG. Proteins were transferred to nitrocellulose membranes which were then incubated in the primary antibody solution overnight, followed by a washing procedure carried out three times within 15 minutes. The nitrocellulose membranes were then incubated with the second antibody solution for one hour at room temperature. The washing procedure was repeated three times within 15 minutes. Immunoreactive bands were visualized by enhanced chemiluminescence (ECL; Amersham Biosciences), which was then exposed to Biomax L film (Kodak). For quantification, ECL signals were digitized using Labwork software (UVP).

### Statistical analysis

All data were presented as mean ± SD. Statistical analyses were performed using SPSS 18.0 (SPSS Inc, Chicago, IL) to conduct one-way and two-way analysis of variance (ANOVA), followed by a Bonferroni multiple-comparison post hoc test to compare the physiological parameters. A Mann–Whitney *U* test was used to compare the severity of WM lesions between the groups. *P* < 0.05 was considered to be statistically significant.
