# Correction: Melatonin attenuated the brain damage and cognitive impairment partially through MT2 melatonin receptor in mice with chronic cerebral hypoperfusion

**DOI:** 10.18632/oncotarget.27754

**Published:** 2020-09-22

**Authors:** Tzu-Hsien Tsai, Cheng-Jei Lin, Sarah Chua, Sheng-Ying Chung, Cheng-Hsu Yang, Meng-Shen Tong, Chi-Ling Hang

**Affiliations:** ^1^ Division of Cardiology, Department of Internal Medicine, Kaohsiung Chang Gung Memorial Hospital and Chang Gung University College of Medicine, Kaohsiung, Taiwan; ^2^ Center for Translational Research in Biomedical Sciences, Kaohsiung Chang Gung Memorial Hospital and Chang Gung University College of Medicine, Kaohsiung, Taiwan

**This article has been corrected:** The co-first author, Cheng-Jei Lin, has been removed as an equal contributor for this study as compared to the first author.

Original article: Oncotarget. 2017; 8:74320–74330. 74320-74330. https://doi.org/10.18632/oncotarget.20382

